# Beneficial Effects of Grape Pomace Extract on Hyperglycemia, Dyslipidemia, and Oxidative Stress in Experimental Diabetes Mellitus

**DOI:** 10.3390/molecules30214183

**Published:** 2025-10-25

**Authors:** Mariya Sabadashka, Dariya Chala, Grzegorz Chrzanowski, Jan Cichoński, Nataliia Sybirna

**Affiliations:** 1 Department of Biochemistry, Faculty of Biology, Ivan Franko National University of Lviv, 1, Universytetska St., 79000 Lviv, Ukraine; dariya.hertsyk@lnu.edu.ua; 2 Collegium Medicum, Faculty of Biotechnology, University of Rzeszow, 8B Zelwerowicza St., 35-601 Rzeszow, Poland; gchrzanowski@ur.edu.pl; 3 Doctoral School, University of Rzeszow, Rejtana 16C, 35-959 Rzeszow, Poland; janc@dokt.ur.edu.pl

**Keywords:** grape pomace, polyphenols, diabetes mellitus, hyperglycemia, dyslipidemia, antioxidant

## Abstract

Grape pomace, a polyphenol-rich byproduct of wine production, represents a promising source of bioactive compounds for managing diabetes and its complications. This study evaluates the effect of a novel grape pomace extract on carbohydrate and lipid metabolism, and oxidative stress in type 1 diabetes mellitus. Diabetes was induced in male Wistar rats by a single intraperitoneal injection of streptozotocin. Starting on day 14 post-induction, rats received oral grape pomace extract at a dose of 45 mg of polyphenols/kg body weight daily for 14 days. On day 28 of the experiment, blood plasma was collected. One-way ANOVA with post hoc testing revealed a hypoglycemic effect of grape pomace extract, as evidenced by reduced fasting blood glucose and improved postprandial glycemic responses. The extract also ameliorated dyslipidemia, lowering total cholesterol and triglycerides while increasing high-density lipoprotein levels and paraoxonase activity in plasma of diabetic rats. Antioxidant defenses were enhanced, as indicated by elevated superoxide dismutase, catalase, and glutathione peroxidase activities, along with reduced protein carbonyls, TBA-reactive products, and lipofuscin in blood plasma following extract administration. These findings demonstrate the metabolic and antioxidant potential of grape pomace polyphenols, although further investigations are needed to elucidate the underlying molecular mechanisms.

## 1. Introduction

Diabetes mellitus is a chronic disease characterized by elevated blood glucose levels, resulting from impaired insulin secretion or action. Impaired carbohydrate, lipid, and protein metabolism, as well as oxidative stress, are key factors contributing to diabetic complications that affect various organs, mainly blood vessels, nerves, kidneys, and eyes [1]. The complex etiology and pathogenesis of diabetes make it difficult to develop effective methods for early diagnosis and treatment. Two main types of diabetes are distinguished: types 1 and 2. Patients with type 2 account for more than 85% of the total diabetes mellitus prevalence. The importance of lifestyle and other modifiable factors in the development of type 2 diabetes is well established, making prevention a realistic public health goal. On the contrary, the etiology of type 1 diabetes remains incompletely understood [2], and thus, it cannot currently be prevented [3].

Epidemiological data confirm that polyphenols and polyphenol-rich products can improve the health of patients with several chronic diseases, including diabetes, obesity, cardiovascular diseases, and certain types of cancer [4,5]. Plant polyphenols have demonstrated antioxidant, antiradical, vasodilating, immunostimulating, antitumoral, anti-aging, anti-inflammatory, anti-allergic, antiviral, antimicrobial, and estrogenic effects both in vivo and in vitro [6,7,8]. Therefore, medicines based on plant raw materials can be recommended as adjuvant therapy in diabetes and as dietary supplements for preventing diabetic complications.

Grape pomace represents a promising source of polyphenols. It is a by-product of winemaking and is rich in bioactive compounds. Large amounts of grape pomace are produced annually, leading to waste management challenges. Currently, its use is mainly limited to processing into organic fertilizers or animal feed. However, grape pomace is a rich source of polyphenols such as anthocyanins, flavanols, catechins, and proanthocyanidins [6,9,10,11]. Therefore, it is a promising raw material for obtaining nutraceuticals rich in polyphenols.

We have developed a new method for obtaining an extract from grape pomace with a high content of polyphenols. The aim of this study was to evaluate the effects of the grape pomace extract on carbohydrate and lipid metabolism, and oxidative stress in type 1 diabetes mellitus, revealing a unique multitarget profile with pronounced hypoglycemic, antidyslipidemic, and antioxidant effects.

## 2. Results

### 2.1. Quantitative and Qualitative Composition of Grape Pomace Extract

Six phenolic acids (catechol, gallic acid, gallic acid hexoside, vanillic acid, p-coumaric acid, and trans-cinnamic acid), nine flavonoids (catechin, epicatechin, rutin, quercetin 3-O-glucoside, quercetin 3-O-rhamnoside, quercetin, naringenin, myricetin, myricetin 3-O-glucoside), and seven anthocyanins (malvidin 3-O-glucoside, peonidin 3-O-glucoside, cyanidin 3-O-glucoside, petunidin 3-O-glucoside, cyanidin 3-O-(6″-O-acetyl)-glucoside, delphinidin 3-O-(6″-O-acetyl)-glucoside, phloretin 2′-glucuronide) were identified in grape pomace extract (Table 1). The identification of the analyzed compounds was made based on retention time of separated metabolites and standards, as well as using the characteristic mass-to-charge ratio (*m*/*z*), and observed ions produced during fragmentation.

### 2.2. Antioxidant Capacity of Grape Pomace Extract In Vitro

**Figure 1:**
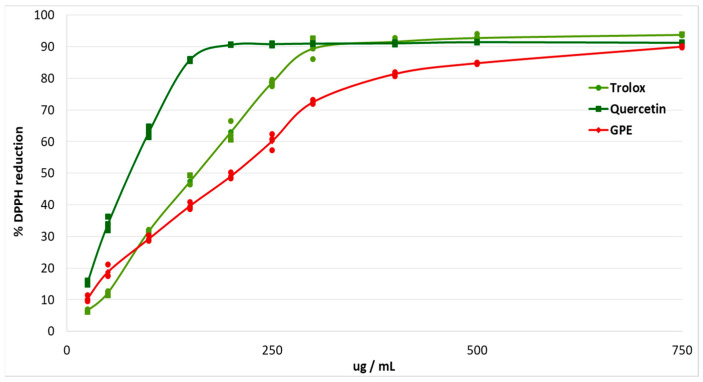
Percentage of DPPH reduction after addition of different concentrations of grape pomace extract (GPE), Trolox and Quercetin.

The antioxidant effects of plant polyphenols are crucial for the protection of the organism from oxidative stress, and may be beneficial for diseases such as diabetes [12]. The study of antioxidant capacity in vitro made it possible to evaluate the ability of grape pomace extract to scavenge free radicals. The antiradical effect was studied using the stable free radical DPPH. Trolox and quercetin were used as antioxidant standards (Figure 1).

The EC_50_ of grape pomace extract was calculated (Table 2). The EC_50_ for grape pomace extract was 1.34 times higher than the EC_50_ for Trolox, and 2.7 times higher than the EC_50_ for Quercetin.

### 2.3. Effect of Grape Pomace Extract on the Glycemic Profile

**Figure 2:**
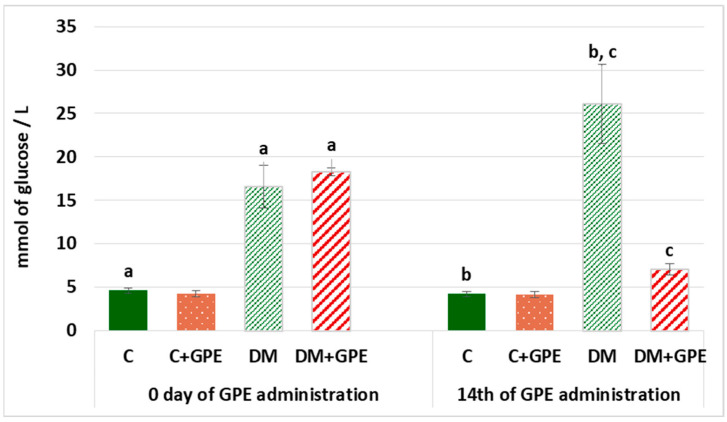
Fasting blood glucose (mmol/L) in control, diabetes mellitus, and after administration of grape pomace extract. Values are arithmetic mean ± standard deviation (*n* = 6). a—data significantly different at *p* < 0.001 on the 1st day of experiment; b, c—data with the same superscript letters are significantly different at *p* < 0.001 on the 14th day of experiment.

Hyperglycemia is one of the main characteristics of diabetes and contributes to the development of diabetic complications. Therefore, it is worth establishing the hypoglycemic potential of bioactive substances. On the first and last days of grape pomace extract administration, fasting blood glucose levels were determined (Figure 2). On the 1st day of the experiment, fasting blood glucose in diabetes exceeded the level of control by 3.6 times, and by the 14th day, it exceeded control values by 6.2 times. Administration of grape pomace extract to diabetic rats for 14 days reduced fasting blood glucose by 3.72 times compared to untreated diabetic group.

**Figure 3:**
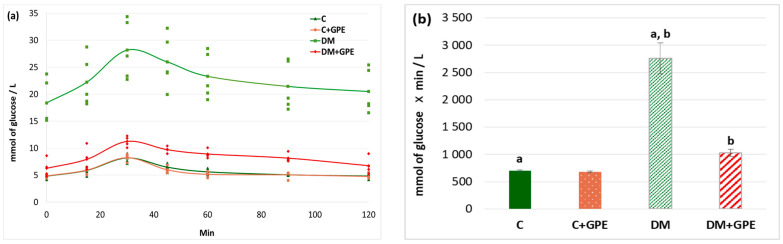
Glycemic curves (mmol/L) after a glucose load (**a**) and the area under glycemic curves (mmol/L) (**b**) in control, diabetes mellitus, and after administration of grape pomace extract. Values are arithmetic mean ± standard deviation (*n* = 5). a, b—data with the same superscript letters are significantly different at *p* < 0.001.

A glucose tolerance test is used for the analysis of the cellular glucose uptake. We found that the maximum blood glucose concentration in diabetic animals occurred at 30 min (28.18 ± 5.44 mmol/L). The glucose concentration began to decrease in diabetic rats at 45 min (25.99 ± 2.49 mmol/L), and by 120 min after the glucose load, it approached the fasting blood glucose level (20.51 ± 2.25 mmol/L) (Figure 3a). In control animals treated with the extract, the glycemic curve did not differ from that of untreated controls (Figure 3a). Under the administration of grape pomace extract to animals with diabetes, the maximum glucose concentration was observed 30 min after glucose load (11.26 ± 0.49 mmol/L), followed by a decrease starting at 45 min (9.73 ± 0.29 mmol/L). By 90 min after glucose load, blood glucose levels in treated diabetic animals approached the fasting blood glucose (8.16 ± 0.42 mmol/L) (Figure 3a).

An increase in the area under glycemic curves by 4-fold was observed in diabetes compared to control. No significant changes in this parameter were found when the grape pomace extract was administered to control animals. In diabetic animals, however, the extract contributed to a 2.7-fold decrease in the area under the curve (Figure 3b).

**Figure 4:**
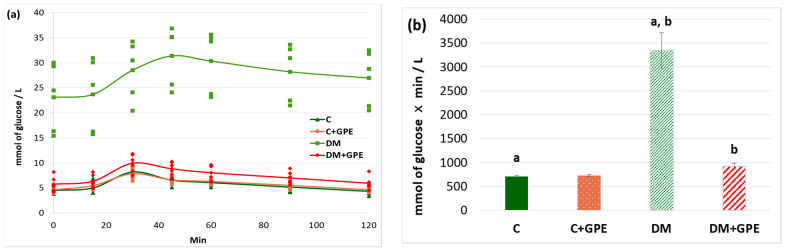
Glycemic curves after a starch load (**a**) and the area under glycemic curves (**b**) in control, diabetes mellitus, and after administration of grape pomace extract. Values are arithmetic mean ± standard deviation (*n* = 6). a, b—data with the same superscript letters are significantly different at *p* < 0.001.

A starch tolerance test was used to evaluate carbohydrate hydrolysis processes in the gastrointestinal tract. The maximum blood glucose concentration in rats with diabetes was recorded 60 min after starch load (30.34 ± 3.03 mmol/L). Starting from 80 min, the glucose concentration decreased (28.21 ± 3.09 mmol/L), but even after 120 min, the concentration did not return to the initial values (26.96 ± 2.99 mmol/L) (Figure 4a). Administration of the extract to control rats did not alter the glycemic curve compared to untreated control (Figure 4a). In diabetic animals treated with grape pomace extract, the maximum glucose concentration occurred at 30 min after starch load (9.92 ± 0.72 mmol/L), similar to that in control animals. Starting from 45 min, the glucose concentration decreased (8.82 ± 0.54 mmol/L), and after 120 min from the moment of starch load, the glucose concentration indicator acquired initial values (5.94 ± 0.52 mmol/L) (Figure 4a).

The area under the glycemic curves after a starch load increased by 4.7-fold in diabetes compared to the control group. No changes were observed when the grape pomace extract was administered to control animals. In diabetic animals, the extract contributed to a 3.6-fold decrease in the area under the glycemic curves (Figure 4b).

**Figure 5:**
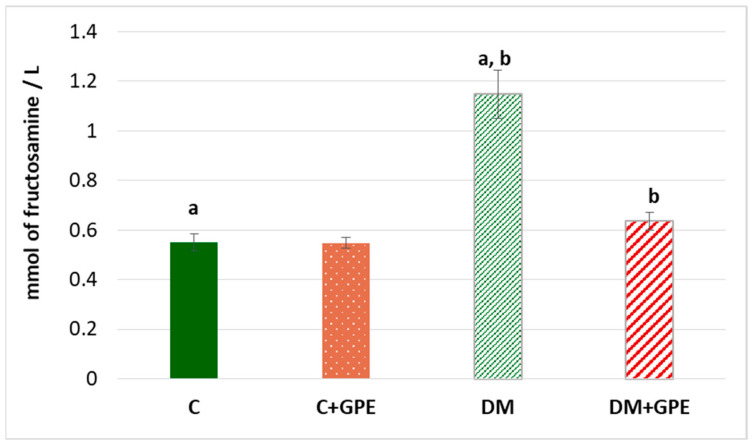
The concentration of fructosamine (mmol/L) in blood plasma in control, diabetes mellitus, and after administration of grape pomace extract. Values are arithmetic mean ± standard deviation (*n* = 10). a, b—data with the same superscript letters are significantly different at *p* < 0.001.

The fructosamine concentration was shown to increase by 2 times in diabetes compared to the control. No significant changes in the parameter were observed when grape pomace extract was administered to control animals. However, in diabetic animals treated with the extract, the concentration of fructosamine decreased by 1.8 times, compared to untreated diabetic animals (Figure 5).

### 2.4. Effect of Grape Pomace Extract on the Lipid Profile

The plasma total cholesterol content increased by 2.1 times in diabetes compared to control (Table 3). Administration of grape pomace extract resulted in a 1.4-fold decrease in total cholesterol observed in diabetic animals.

A 1.9-fold increase in plasma triglyceride content was established in diabetes compared to the control. Administration of grape pomace extract to diabetic animals resulted in normalization of the triglyceride levels to control values (Table 3). We have shown that the plasma HDL content decreased by 1.6 times in diabetes compared to control. Under grape pomace extract administration, HDL levels increased to the normal range in animals with diabetes (Table 3).

**Figure 6:**
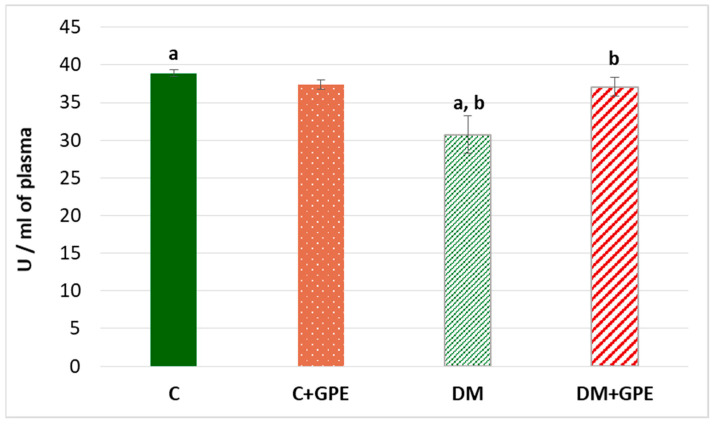
The activity of paraoxonase (U/mL) in blood plasma in control, diabetes mellitus, and after administration of grape pomace extract. Values are arithmetic mean ± standard deviation (*n* = 6). a—data are significantly different at *p* < 0.001; b—data are significantly different at *p* < 0.05.

A 1.3-fold decrease in the activity of paraoxonase was established in the plasma of diabetic animals compared to the control (Figure 6). When the extract from grape pomace was administered, paraoxonase activity increased by 1.2 times in diabetic animals (Figure 6).

### 2.5. Effect of Grape Pomace Extract on Antioxidant System in Blood Plasma in Diabetes

To evaluate the antioxidant effect of the grape pomace extract, we analyzed changes in the activities of superoxide dismutase, catalase, and glutathione peroxidase in blood plasma, which are summarized in Table 4.

We observed a decrease of 1.4 times in both superoxide dismutase and catalase activities in diabetes compared to control group. Administration of grape pomace extract increased superoxide dismutase activity by 1.4 times and catalase activity by 1.5 times in diabetic animals compared to untreated diabetes (Table 4). We have shown a decrease in glutathione peroxidase activity by 1.35 times in diabetes. Administration of grape pomace extract was accompanied by an increase in enzyme activity by 1.3 times in the plasma of diabetic animals (Table 4).

In addition to altered antioxidant enzyme activities, increased levels of protein oxidation and lipid peroxidation are important markers of oxidative stress in diabetes.

**Figure 7:**
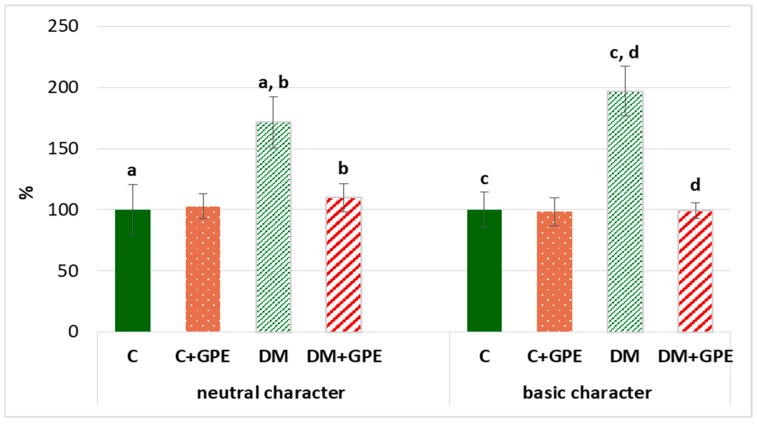
The content of proteins oxidative modification products of neutral (370 nm) and basic (430 nm) character in plasma in control, diabetes mellitus, and after administration of grape pomace extract. The results are shown in % (the value of control animals was considered 100%). Values are arithmetic mean ± standard deviation (*n* = 5). a, b—data on the content of proteins oxidative modification products of neutral character with the same superscript letters are significantly different at *p* < 0.05; c, d—data on the content of proteins oxidative modification products of basic character with the same superscript letters are significantly different at *p* < 0.001.

We found that the content of carbonyl derivatives of proteins of a neutral and basic character increased by 72% and 97%, respectively, in plasma in diabetes, compared to the control. Treatment with grape pomace extract led to a decrease of carbonyl derivatives of neutral and basic character by 62% and 98%, respectively, in diabetic rats (Figure 7).

**Figure 8:**
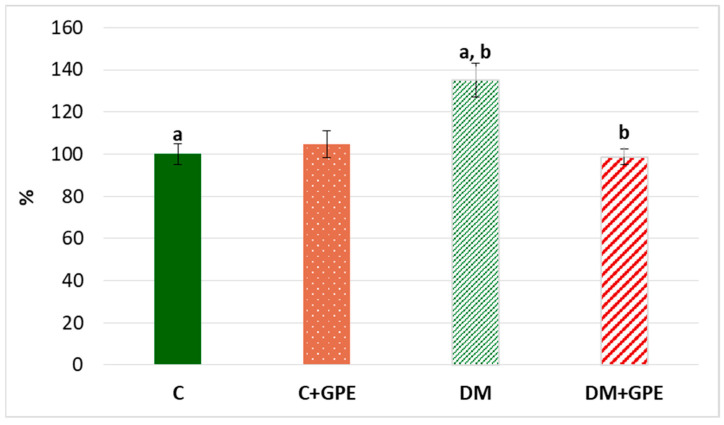
The content of TBA-reactive products in plasma in control, diabetes mellitus, and after administration of grape pomace extract. The results are shown in % (the value of control animals was considered 100%). Values are arithmetic mean ± standard deviation (*n* = 5). a, b—data with the same superscript letters are significantly different at *p* < 0.01.

We observed a 35% increase in the content of TBA-reactive products in plasma in diabetes compared to the control (Figure 8). Administration of grape pomace extract contributed to a 36% decrease in the content of TBA-reactive products in plasma of diabetic animals compared to untreated diabetes.

**Figure 9:**
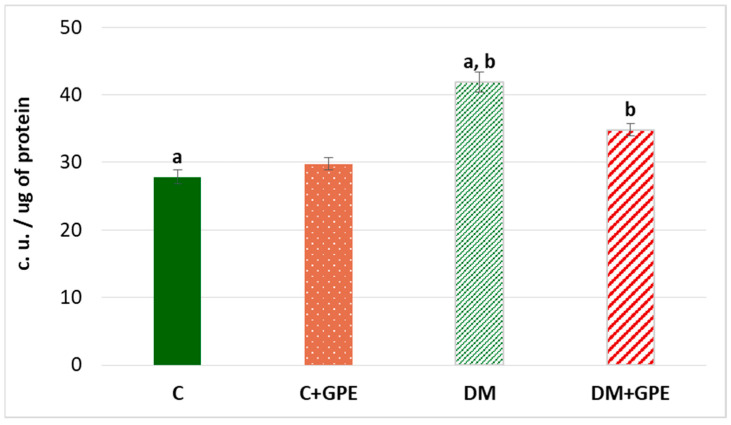
The content of lipofuscin (c.u./ug) in plasma in control, diabetes mellitus, and after administration of grape pomace extract. Values are arithmetic mean ± standard deviation (*n* = 6). a, b—data with the same superscript letters are significantly different at *p* < 0.001.

Oxidative stress is accompanied by an increase in the level of lipofuscin—a complex compound of oxidized proteins and lipids, which is a product of lysosomal digestion. Lipofuscin is synthesized in various cells and organs, from where it can enter the bloodstream [13]. An increase of 50.32% in plasma lipofuscin content was established in experimental diabetes compared to control group. Administration of grape pomace extract to diabetic animals led to a reduction in plasma lipofuscin content by 25.47% compared to diabetes, but the level remained higher than in the control group (Figure 9).

## 3. Discussion

The present study provides evidence of the beneficial biological activity of grape pomace extract, encompassing its chemical composition, in vitro antiradical properties, and in vivo effects on metabolic and oxidative parameters in experimental diabetes.

The extract was characterized by a rich quantitative and qualitative profile of polyphenolic compounds, which are widely recognized for their bioactive potential. The presence of six phenolic acids, nine flavonoids, and seven anthocyanins in the extract was confirmed. Moreover, the results of the analysis were compared with the literature data [14,15,16,17]. Although grape pomace contains twenty times fewer anthocyanins than grape skins analyzed by Kapusta et al. [14], our results suggest that it could be an important source of active metabolites. This is particularly significant considering the large quantity of pomace generated during wine production. On the other hand, as reported by Czerniewicz et al. [18], a high amount of flavonoids, particularly catechins, may increase antioxidative capacity and reduce superoxide anion radical content. Previously, we revealed biochemical and molecular mechanisms of action of polyphenol concentrate from grape wine [19]. When comparing EC_50_ values, higher antioxidant activity was demonstrated for the grape pomace extract compared to the wine concentrate. This result may be associated with a higher content of anthocyanins in the studied extract.

Literature data indicate that polyphenols such as flavonoids and phenolic acids undergo extensive intestinal and hepatic metabolism (glucuronidation and/or sulfation) after oral administration, and repeated dosing leads to the accumulation of conjugated metabolites in various tissues, notably in liver and muscle [20]. Some anthocyanins (including malvidin, cyanidin, and peonidin derivatives) are absorbed either intact or as conjugates. Importantly, the gut microbiota generates small phenolic acids that are highly bioavailable [21,22]. With repeated administration, polyphenols exhibit increasing plasma and tissue levels, reaching higher steady-state concentrations over days to weeks, with reports of peak accumulation observed around 14 days in rodents [23,24]. In our study, grape pomace extract was administered to control and diabetic rats for 14 days, which is consistent with the reported accumulation of mentioned polyphenols and their metabolites in plasma and tissues. This may help explain the observed changes in functional parameters (glucose tolerance, enzyme activities) after two weeks of treatment.

We confirmed the hypoglycemic effect of the grape pomace extract by a reduction in fasting blood glucose. We can conclude that polyphenols lower blood glucose concentration by enhancing cells glucose uptake, as confirmed by glucose tolerance tests and literature data [25,26,27]. It was reported that quercetin and its glycosides stimulate GLUT4 translocation and expression in muscle and adipose cells, thereby promoting glucose uptake [28,29]. In addition, the results of the starch tolerance test indicate that the extract may influence glucose absorption in the intestine, which aligns with previous findings [30,31,32,33]. Catechin and epicatechin have also been shown to inhibit α-glucosidase and α-amylase activity in vitro, which would slow digestion of starches and flatten postprandial glucose peaks [29,31]. It is worth noting that the extract affects the studied parameters only under hyperglycemic conditions; no changes were observed in glucose or starch load test results in control animals treated with the extract.

Violation of carbohydrate metabolism in diabetes can lead to the accumulation of protein glycation products, known as advanced glycation end products (AGEs). These products form through the irreversible addition of reducing sugars to free amino groups of proteins [34]. Fructosamine is among AGEs formed by glycation of blood plasma proteins, particularly albumin, globulins, and lipoproteins [35]. An increase in fructosamine concentration was observed in the blood plasma of diabetic rats, whereas administration of grape pomace extract to diabetic rats almost restored levels to those of the control group. This suggests that the extract possesses a pronounced hypoglycemic effect, which is evident even after short-term administration in type 1 diabetes.

Dyslipidemia in diabetes is characterized by elevated total cholesterol and triglycerides levels, along with decreased HDL content. It represents an important metabolic consequence of the disease and may appear long before hyperglycemia develops in patients with diabetes [36,37]. Our results indicate that grape pomace extract contributed to the reduction in total cholesterol and triglycerides levels while increasing the HDL content in the plasma of diabetic rats. These effects may be explained by multiple mechanisms through which grape polyphenols influence lipid metabolism, including reduced intestinal cholesterol absorption and decreased hepatic transport of cholesterol, ultimately lowering plasma cholesterol levels [4,38].

Deterioration of glycemic control in patients with diabetes contributes to elevated blood triglycerides due to increased synthesis in the liver [39]. The biological mechanisms underlying the positive effect of grape pomace extract on free triglyceride levels involve the inhibition of triglyceride synthesis. Along with this, polyphenols also inhibit cholesterol synthesis. In particular, quercetin and myricetin have been shown to downregulate hepatic expression of 3-hydroxy-3-methylglutaryl-CoA reductase and transcription factor SREBP-1c, thereby reducing de novo cholesterol and triglyceride synthesis [40]. Polyphenols also can inhibit pancreatic lipase, suppressing triglyceride secretion, and may reduce the synthesis of apolipoprotein-B (Apo-B), the main protein component of HDL [41]. In a physiological state, HDL exhibits anti-atherogenic properties, such as facilitating cholesterol transport and providing antioxidant and anti-inflammatory effects. However, in diabetes, these functions are impaired due to oxidative modification and glycation of HDL proteins [42]. The increase in HDL cholesterol following grape pomace extract administration may be associated with the hypoglycemic and anti-inflammatory effects of polyphenols [43]. Additionally, polyphenols like quercetin and resveratrol can enhance HDL functionality by improving cholesterol efflux through increased expression of ABCG1 and ABCA1 transporters [44].

Paraoxonase activity was investigated. Paraoxonase plays an important role in the inhibition of LDL and HDL oxidation. It is mainly expressed in the liver and secreted into the blood, where it binds to HDL. A decrease in paraoxonase activity was observed in the blood plasma of diabetic rats, which is consistent with literature data [45,46]. Under hyperglycemic conditions, both gene expression and paraoxonase activity are affected. In diabetes, several factors associated with high glucose, dyslipidemia, and oxidative stress may contribute to reduced paraoxonase activity. Among these factors is the non-enzymatic glycosylation of paraoxonase, which inhibits the enzyme [46].

Administration of grape pomace extract resulted in a significant elevation of paraoxonase activity. Polyphenolic compounds, such as quercetin and resveratrol, can modulate paraoxonase regulation. One likely mechanism is the activation of AhR (aryl hydrocarbon receptor) by polyphenols, which induces paraoxonase activation. Inflammation is also known to negatively affect paraoxonase. Activation of NF-kB stimulates the expression of genes, including cytokines and interleukins (in particular IL-1, IL-6, TNF-α), which can inhibit paraoxonase. Polyphenolic compounds prevent NF-κB activation by inhibiting IκB phosphorylation, thereby suppressing the inflammatory cascade and restoring enzyme activity [47].

The effect of grape pomace extract on the prooxidant–antioxidant balance in the blood plasma of rats with type 1 diabetes mellitus was further examined. The antioxidant capacity of plasma is a key indicator for assessing the level of oxidative stress in vivo. Plasma contains numerous compounds with antioxidant activity that protect cells and biomolecules from damage. The combined action of all antioxidant molecules ensures the antioxidant capacity of the plasma [48].

A decrease in the activities of superoxide dismutase, catalase, and gluthatione peroxidase was observed under diabetic conditions, which may be explained by hyperglycemia-related non-enzymatic glycosylation leading to enzyme inhibition. Moreover, the excessive formation of ROS, such as superoxide, hydroxyl radicals, and hydrogen peroxide, can inhibit these through a negative feedback mechanism [49,50,51]. As mentioned earlier, we observed a reduction in glucose levels when grape pomace extract was administered to diabetic rats and the anti-radical effect of the extract in vitro. The increase in glutathione peroxidase activity after administration of the extract may be related to the ability of polyphenols to modulate the concentration of endogenous glutathione, which mediates enzyme activation [52].

The enhanced activities of antioxidant enzymes following grape pomace extract administration confirm the involvement of polyphenols in protection against oxidative stress. The antioxidant action of polyphenols may be attributed to the presence of functional hydroxyl (OH) groups, which inhibit ROS synthesis, chelate metal ions responsible for free radical formation, and scavenge ROS [53]. In addition, grape polyphenols can enhance the activities of antioxidant enzymes. This may be due to the activation of the transcription factor Nrf2 (nuclear factor erythroid 2-related factor 2) and upregulation of genes encoding antioxidant enzymes such as superoxide dismutase, catalase, and glutathione peroxidase [54].

Protein conformation and function are affected by modification resulting from interaction with ROS. Carbonyl protein derivatives are formed due to oxidative modification of proteins, particularly oxidative deamination of amino acids (lysine, threonine, arginine, proline, and others) [48,55]. Such modifications often lead to loss of protein function, thereby disrupting regulation of metabolic pathways. Since protein carbonylation is irreversible oxidative damage, the modified proteins are subsequently degraded by the cellular proteasome system [13,56].

Diabetes mellitus is accompanied by disturbance in the lipid profile, which increases cellular susceptibility to lipid peroxidation (LPO). ROS can react with polyunsaturated fatty acids (PUFAs) in the LPO process, whereby PUFAs react with molecular oxygen to form lipid hydroperoxides as primary products and aldehydes (such as malondialdehyde, 4-hydroxynonenal, and 4-oxononenal) as end products [48,57].

Among the many benefits of polyphenol compounds, their ability to inhibit oxidation stands out. Polyphenols exhibit antioxidant activity by scavenging free radicals and chelating metal ions (for example, quercetin chelates Fe^2+^ ions) [52]. In particular, anthocyanins, abundant in brightly colored fruits—especially grapes—possess strong antioxidant and anti-inflammatory properties, suppress lipid peroxidation and protein oxidation, and interrupt oxidative chain reactions. Through these mechanisms, polyphenols preserve the structure and functionality of cellular molecules [58] and contribute to decreased lipofuscin levels.

The present study provides a comprehensive evaluation of the biological activity of grape pomace extract in experimental type 1 diabetes through an integrative approach combining chemical characterization, in vitro antioxidant assays, and in vivo assessments of metabolic and oxidative stress parameters. This multifaceted analysis demonstrates that grape pomace extract exerts coordinated effects on carbohydrate and lipid metabolism, antioxidant enzyme activity, and oxidative damage markers, supporting its distinctive multi-target profile with potent hypoglycemic, antidyslipidemic, and antioxidant actions. These findings highlight grape pomace as a promising source of bioactive polyphenols with therapeutic potential in diabetes management. Future studies should aim to elucidate the molecular mechanisms underlying these effects, with particular focus on signaling pathways involved in glucose uptake (e.g., GLUT4), energy homeostasis (e.g., AMPK), and redox regulation (e.g., SOD, catalase). Such investigations will be essential to confirm the mechanistic basis and translational relevance of grape pomace polyphenols in metabolic disease.

## 4. Materials and Methods

### 4.1. Chemicals and Standards

Streptozotocin (S0130; purity ≥98%), gallic acid (3,4,5-trihydroxybenzoic acid monohydrate; 398225; ACS reagent; ≥98%), 2,2′-bis(4-Nitrophenyl)−5,5′-diphenyl-3,3′-(3,3′-dimethoxy-4,4′-diphenylene) ditetrazolium chloride (nitrotetrazolium blue chloride, NTB; 93862; purity ≥90%), 1-deoxy-1-morpholinofructose (D6149; purity ≥98%), bovine serum albumin (BSAV-RO; purity ≥98.5%), reagents for phenolic compound determination: Folin–Ciocalteau reagent (F9252), sodium carbonate (Na_2_CO_3_; 223530; ACS reagent; ≥99.5%), sodium nitrite (NaNO_2_; 237213; ACS reagent, ≥97.0%), and aluminum chloride (AlCl_3_; 206911; purity ≥98%) were purchased from Sigma-Aldrich (Poznan, Poland). Acetonitrile (1.00029; LC-MS grade; ≥99.9% (GC)) and formic acid (1.59013; LC-MS grade) were purchased from Sigma Aldrich (Poznan, Poland). Standards for phenolic acids and flavonoid separation were obtained from Sigma-Aldrich (Poznan, Poland), while standards for anthocyanins were purchased from Extrasynthese (Lyon, France). Commercially available kits for glucose (HP009.02) concentration, triglycerides (HP022.04), total cholesterol (HP026.10), and HDL cholesterol (HP026.04) content measurement were purchased from Filisit Diagnostica (Kyiv, Ukraine). Other chemical reagents were produced in Ukraine. All reagents were of analytical grade.

### 4.2. Extract from Grape Pomace Obtaining

Grape pomace was obtained after the production of dry grape wine from Odesa black grapes, the characteristics of which were described previously [19]. The extract was prepared according to the technology newly developed by the authors [59]. Crushed dry grape pomace was incubated with 50% ethanol acidified with citric acid at 50 °C for 1 h, and the resulting mixture was filtered. The solid fraction was secondarily incubated with 96% ethanol acidified with citric acid at 50 °C for 1 h. After incubation, the mixture was filtered and centrifuged for 5 min at 3000 rpm to precipitate the solid fraction. The liquid fractions were evaporated on a Laborota 4001 rotary evaporator (Heidolph Instruments, Schwabach, Germany, ) at 40 °C and a pressure of 0.8–0.9 kg/cm^2^.

### 4.3. Qualitative and Quantitative Determination of Polyphenols in the Grape Pomace Extract

The extract was diluted with 50% ethanol, and then total phenols and flavonoids were determined by spectrophotometric method, whereas individual constituents of phenolic acids, flavonoids, and anthocyanins were determined with liquid chromatography.

Spectrophotometric determination. Total polyphenol content was determined using the Folin–Ciocalteu′s phenol reagent (Sigma-Aldrich, Poznan, Poland), whereas total flavonoid content was analyzed with the aluminum chloride reagent. Both methods were carried out according to protocols described by Jańczak-Pieniążek et al. [60].

Chromatographic analysis. Ultra-high performance liquid chromatography coupled with mass spectrometry (UHPLC-MS) was performed using an ACQUITY system and Synapt G2 mass spectrometer with hybrid quadrupole and time-of-flight analyzer (Waters Corp., Milford, MA, USA). Polyphenolic compounds were separated using an ACQUITY UPLC BEH C18 Column, 130 Å, 1.7 µm, 2.1 mm × 100 mm column (Waters Corp., Milford, MA, USA). The column temperature was set at 45 °C, the autosampler was maintained at 20 °C, and the injection volume was 3 µL. The mobile phases for UPLC-MS were solvent A (0.1% formic acid in water) and solvent B (40% acetonitrile containing 0.1% formic acid) at a flow rate of 0.35 mL × min^−1^. The linear gradient elution was used: 0 min, 5% B; 10 min, 90% B; 12 min, 90% B; back to 5% in 0.1 min. A 1.9 min equilibration time was used between injections. The analysis was performed using electrospray ionization in positive ion mode (ESI+) and negative (ESI−). The optimized MS parameters were as follows: source temperature of 350 °C, desolvation temperature of 120 °C, cone gas flow of 50 L/h, desolvation gas flow of 550 L/h, capillary voltage of 3.6 kV, and cone voltage of 30 V. The MS analysis was performed using a scan range from 150 to 900 *m*/*z*. MassLynx 4.1 software (Waters, USA) was used for instrument control and data analysis.

All analyses were performed in three independent repetitions, and the results for the polyphenols were expressed as the mean value ± standard deviation.

### 4.4. DPPH Scavenging Assay

The antioxidative capacity of grape pomace extract was determined by the percentage of reduction of 2, 2-diphenyl-1-picrylhydrazyl radical (DPPH) [61]. Trolox (6-hydroxy-2,5,7,8-tetramethyl chroman-2-carboxylic acid) and quercetin were used as standards. We measured the antioxidant capacity of the following concentrations of standards (μg/mL): 0, 12.5, 25, 50, 75, 100, 125, 150, 175, 200. We prepared a series of dilutions of grape pomace extract containing the following concentrations of polyphenolic compounds (μg/mL): 0, 12.5, 25, 50, 75, 100, 125, 150, 175, 200.

1.45 mL of 0.1 mM DPPH solution in methanol, prepared immediately before usage, was added to 0.05 mL of sample and standards. After 30 min of incubation at room temperature in the dark, absorbance was measured at a wavelength of 517 nm. The percentage of DPPH reduction was calculated using Formula (1).

% DPPH reduction = (A_0_ − A_1_) × 100/A_0_, (1)

where A_0_ stands for the value of light absorption of DPPH solution without the presence of antioxidants (control), A_1_ stands for the value of light absorption of DPPH solution containing the antioxidants (experiment).

The concentration of grape pomace extract providing a 50% reduction in DPPH (EC_50_) was calculated from the graph plotting the percentage of reduced DPPH against the sample concentration.

### 4.5. Animals

The experiments were conducted following the General Principles of Animal Treatment approved by the First National Congress on Bioethics (Kyiv, Ukraine, 2001) and agreed with the guidelines of Directive 2010/63/EU of the European Parliament and of the Council on the protection of animals used for scientific purposes and the Law of Ukraine “On Protection of Animals from Cruelty” of 26 February 2006, and approved by the Ethics Committee of Ivan Franko National University of Lviv, Ukraine (protocol No. 46–11-2024 from 4 November 2024). Rats were kept in the vivarium of the Faculty of Biology of Ivan Franko National University of Lviv, Ukraine. Rats were housed in cages at a controlled temperature ranging from 21 to 25 °C. A twelve-hour day–night cycle and ad libitum food and water were maintained.

### 4.6. Induction of Diabetes Mellitus in Rats

The animals were single injected with streptozotocin at the dose of 60 mg/kg of body weight intraperitoneally after 18 h of starvation. Streptozotocin immediately before the injection was dissolved in 10 mM citrate buffer (рН 5.5). The STZ protocol used induces rapid β-cell cytotoxicity and results in a model that resembles insulin-deficient diabetes, broadly comparable to type 1 diabetes mellitus [62].

Fasting blood glucose was measured on the 3rd and 14th days after streptozotocin injection. Glucose in the blood of diabetic rats exceeded 12 mM.

Induction of type 1 diabetes using this streptozotocin protocol has been previously confirmed. We have demonstrated a decrease in insulin and C-peptide levels in the blood of rats with streptozotocin-induced diabetes. Diabetes development was associated with damage to the pancreatic endocrine apparatus, including an uneven distribution of the islets of Langerhans and, in some areas, their complete absence [63].

### 4.7. Determination of Glucose Concentration

The glucose concentration was measured using the glucose oxidase method using a commercially available kit (Filisit diagnostica, Kyiv, Ukraine). In the glucose oxidase reaction, glucose reacts with atmospheric oxygen to form gluconic acid and hydrogen peroxide. In the peroxidase reaction, hydrogen peroxide reacts with phenol and 4-aminophenazone to form a red/violet quinone imine.

To 0.1 mL of capillary blood, 0.9 mL of anticoagulant solution was added. The mixture was centrifuged for 5 min at 3000 rpm to sediment erythrocytes. To 0.1 mL of the supernatant, 0.5 mL of the buffer (containing 0.1 mmol/L phosphate buffer (pH 7.2–7.4), 190 mg/L of phenol, and stabilizers) was added. The mixture was mixed thoroughly, and 0.5 mL of the enzymes solution (containing 2200 U/L peroxidase, 18,000 U/L beta, D-glucose oxidase, 110 mg/L 4-aminophenazone, stabilizers, and activators) was added immediately. The mixture was incubated for 12 min at 37 °C.

The absorbance of the samples was measured at 550 nm. The results were calculated using Formula (2).

C = Es × 10/Ecal,(2)

where C stands for the glucose concentration in the sample, mmol/L, Es stands for the value of light absorption of the sample; 10 stands for the glucose concentration in the control sample (mmol/L), Ecal stands for the value of light absorption of the control sample.

### 4.8. Administration of Grape Pomace Extract to Animals

The grape pomace extract was administered orally with water using a probe. The total volume of the administered preparation was 1 mL. The amount of extract contained in the administered solution was proportional to the weight of each animal. For animals with diabetes mellitus, the extract was administered starting from the 14th day after streptozotocin injection. The administered aliquot of the preparation provided a dose of 45 mg of polyphenolic compounds per 1 kg of animal body weight.

This dose was selected based on data from the literature and our previous findings. In earlier studies, we investigated the biological effects of red wine concentrate [19], which was administered to rats at a dose of 45 mg/kg body weight for 14 days. This dose corresponds to the average concentration of polyphenols found in 300 mL of red wine, which is considered a typical daily intake for a person weighing 70 kg.

### 4.9. Experimental Design

The current research was executed on 48 adult male Wistar rats, 150–180 g, which is equivalent to 6–8 weeks of age. Rats were randomly divided into four groups. Group 1 included non-diabetic control animals (hereinafter called C); Group 2—non-diabetic animals that were per os administered grape pomace extract (hereinafter called C + GPE); Group 3—diabetic animals (hereinafter called DM); and Group 4—diabetic animals that were per os administered grape pomace extract (hereinafter called DM + GPE). Each group included 8–10 rats and was kept in cages separately.

### 4.10. The Glucose Tolerance Test and the Starch Tolerance Test

The oral glucose tolerance test (OGTT) was performed after 18 h of starvation. Fasting glucose (0 min) blood samples were collected from the tail vein of rats. Glucose loading was carried out by oral administration of glucose solution at the dose of 1 g/kg of body weight to animals of each experimental group. Blood samples were then taken at 15, 30, 45, 60, 90, and 120 min after glucose loading. The oral starch tolerance test (OSTT) was performed using the same procedure. Starch loading was carried out by oral administration of starch suspension at the rate of 2 g/kg of body weight. Glucose concentration was measured as described in paragraph 2.7.

The index of the area under the glycemic curve (AUCglu) was calculated by the trapezoid rule [64].

### 4.11. Blood Plasma Collection

The rats from all experimental groups were entered into the surgical stage by ether anesthesia on the 29th day of the experiment. Blood samples were collected from neck vessels immediately after animal sacrifice by decapitation. The blood was collected into a sterile porcelain cup and stirred gently to prevent clotting. Heparin was used as an anticoagulant, and a final dilution of heparin to whole blood was 1:100. The collected blood was placed in tubes and centrifuged for 15 min at 3000 rpm at a temperature of 4 °C to separate plasma. The plasma was carefully removed from the sediment after centrifugation.

### 4.12. Determination of Fructosamine Concentration

Fructosamine concentration was determined by the method described in [65]. To 0.2 mL of carbonate buffer (0.1 mol/L, pH 10.8) containing 0.25 mM nitrotetrazolium blue (NTB), 0.02 mL of blood plasma was added. The mixture was incubated at 37 °C. The absorbance was measured at 530 nm after 10 and 15 min of incubation. The difference between the values after 10 and 15 min of incubation was determined. The fructosamine concentration was determined using the calibration curve. Standard solutions of 1-deoxy-1-morpholinofructose with known concentrations in 0.14 M NaCl plus albumin (40 g/L) were used. The results were expressed in mmol/L.

### 4.13. Determination of Plasma Lipid Profile

#### 4.13.1. Determination of Cholesterol Content

Cholesterol content in blood plasma was determined by a choline esterase–peroxidase coupled enzyme procedure using the detection kit (Filisit diagnostica, Kyiv, Ukraine). The test sample was prepared by the addition of 1 mL of enzyme reagent (containing 150 U/L choline esterase, 100 U/L cholesterol oxidase, 5,000 U/L peroxidase, 0.3 mmol/L 4-aminophenazone, 30 mmol/L phenol, and 30 mmol/L Tris) and 0.01 mL of plasma. The standard sample contained 1 mL of enzyme reagent and 0.01 mL of 5.17 mmol/L cholesterol. The solution in the test tubes was mixed and incubated at 37 °C for 10 min. The absorbance of the samples was measured at 500 nm. The results were expressed in mmol/L.

#### 4.13.2. Determination of Triglyceride Content

The content of triglycerides in the plasma was determined by an enzymatic method using the corresponding detection kit (Filisit diagnostica, Kyiv, Ukraine). The test sample was prepared by the addition of 1 mL of enzyme reagent (containing 40 mmol/L PIPES, pH 7.5, 5 mmol/L 4-chlorophenol, 1 mmol/L MgSO_4_, 0.5 mmol/L 4-aminophenazone, 1500 × 10^6^ U/L lipase, 200 × 10^6^ U/L glycerol kinase, 1000 × 10^6^ U/L glycerol phosphate oxidase, and 250 × 10^6^ U/L peroxidase) and 0.01 mL of plasma. The standard sample contained 1 mL of enzyme reagent and 0.01 mL of 2.26 mmol/L triglycerides. The solution in the test tubes was mixed and incubated at 37 °C for 10 min. The absorbance of the samples was measured at 505 nm. The results were expressed in mmol/L.

#### 4.13.3. Determination of HDL (High-Density Lipoprotein) Cholesterol Content

Chylomicrons, very-low-density lipoproteins (VLDL), and low-density lipoproteins (LDL) were precipitated by the addition of phosphotungstic acid magnesium ions. The content of HDL cholesterol in the plasma was determined by an enzymatic method using the detection kit for the determination of cholesterol content (see Section 4.13.1).

### 4.14. Determination of Paraoxonase Activity

Paraoxonase activity was determined by the rate of 4-nitrophenol formation [66]. To 0.44 mL of basal buffer (0.625 mM 4-nitrophenyl acetate, 10 mM CaCl_2_, and 25% methanol in 25 mM Tris-HCl buffer, pH 7.4), 0.01 mL of plasma was added. The absorbance of the samples was measured at 402 nm. The results were expressed in U/L.

### 4.15. Determination of Antioxidant Enzymes Activity

#### 4.15.1. Determination of Superoxide Dismutase Activity

The method is based on the ability of superoxide dismutase to compete with nitrotetrazolium blue for superoxide anions. In the samples, superoxide anions are formed in the reaction of NADH with phenazine methosulfate [19]. To 0.1 mL of plasma were added 0.9 mL of distilled water, 0.5 mL of absolute alcohol, 0.25 mL of chloroform, and 300 mg of KH_2_PO_4_. The mixture was shaken for 5 min and centrifuged at 2500 g for 30 min. The incubation solution (containing 39 mM EDTA-Na, 114 mM nitrotetrazolium blue [meta form], 54 mM phenazine metasulfate in 0.15 M phosphate buffer, pH 7.8) with 10 μL of NADH, was added to the samples. The reaction was carried out at 24–25 °C for 10 min. The absorbance was measured at 540 nm. The obtained absorbance values were calculated to International Units of Activity (U) using a calibration curve. Superoxide dismutase activity was expressed in U per 1 mg of protein.

#### 4.15.2. Determination of Catalase Activity

Catalase activity was measured by the amount of the colored complex formed between H_2_O_2_ and ammonium molybdate [19]. Catalase decomposes hydrogen peroxide, thereby reducing the intensity of the color in the sample. To 0.1 mL of blood plasma, 2 mL of 0.03% H_2_O_2_ was added. After incubation at 37 °C for 10 min, the reaction was stopped by adding 1 mL of 0.125 M H_2_SO_4_. Then, 1 mL of 4% (NH_4_)_2_MoO_4_ was added to the samples. The samples were centrifuged at 10,000 rpm for 10 min. Color intensity was determined spectrophotometrically at 410 nm. The results were expressed in nmol H_2_O_2_ per min per mg of protein.

#### 4.15.3. Determination of the Activity of Glutathione Peroxidase

The method is based on determining the rate of oxidation of reduced glutathione (GSH) in the glutathione peroxidase reaction [19]. The level of reduced glutathione is measured by the decrease in the color of the sample. The color reaction is based on the formation of the thionitrophenyl anion as a result of the interaction of the SH groups of GSH with 5′-Dithiobis(2-nitrobenzoic acid). A 0.1 mL blood sample was incubated at 37 °C for 10 min with 0.83 mL of 0.1 M Tris-HCl buffer (pH 8.5), containing 6 mM EDTA, 12 mM sodium azide (NaN_3_), and 4.8 mM reduced glutathione (GSH). Then, 0.07 mL of 20 mM tert-butyl hydroperoxide was added and incubated for 5 min. The reaction was stopped by adding 20% trichloroacetic acid (TCA). Samples were centrifuged at 10,000 rpm for 10 min. To 0.02 mL of the supernatant, an equal volume of Ellman’s reagent (0.01 M 5,5’-dithiobis-2-nitrobenzoic acid diluted in methanol) and 2 mL of 0.1 M Tris-HCl buffer (pH 8.5) were added. After 5 min incubation, absorbance was measured at 412 nm. The results were expressed in nmol GSH for 1 min per 1 mg protein.

### 4.16. Determination of Protein Concentration by the Lowry Method

Protein concentration was determined by the Lowry method using the Folin–Ciocalteu reagent [67]. Reagent “A” (containing 20 g of Na_2_CO_3_ dissolved in 1 L of 0.1 M NaOH) and reagent “B” (containing 10 g of sodium citrate and 5 g of CuSO_4_ × 5H_2_O dissolved in 1 L of H_2_O) were prepared. Reagent “C” was prepared immediately before using by mixing 50 mL of reagent “A” and 1 mL of reagent “B”.

To 0.4 mL of the sample, 2 mL of reagent “C” was added. The mixture was stirred, and after 10 min 0.2 mL of Folin–Ciocalteu reagent was added. After 30 min of incubation in the dark at 20 °C, light absorption was measured at 750 nm. The protein concentration was determined using the calibration curve. The results were expressed in mg per mL.

### 4.17. Determination of the Content of the Products of Oxidative Modification of Proteins

Aldehyde or ketone groups are formed in the side chain of amino acids in the process of protein oxidation. These groups interact with 2,4-dinitrophenylhydrazine, resulting in the formation of 2,4-dinitrophenylhydrazones, which have a characteristic absorption spectrum. The determination was carried out according to the procedure described earlier [19].

To 0.2 mL of blood plasma, 0.8 mL of 0.85% NaCl, 1 mL of 0.1 M 2,4-dinitrophenylhydrazine dissolved in 2 M HCl, and 1 mL of 10% TCA were added. Samples were incubated for 1 h at 37 °C with further centrifugation for 10 min at 3000 rpm. The precipitate was washed three times with 5% TCA. Afterward, the precipitate was incubated for 5 min with 5 mL of 8 M urea in a boiling water bath until complete dissolution. The light absorption of the formed dinitrophenylhydrazones complexes was measured at 370 nm (to determine the level of products of neutral character) and 430 nm (to determine the level of products of basic character). The content of phenylhydrazones of neutral character (370 nm) was calculated using the molar extinction coefficient ε = 22,000 per cm per M and expressed in mmol per 1 mL of plasma. The content of phenylhydrazones of basic character (430 nm) was calculated in conventional units (c. u.) per 1 mL of plasma. The results were recalculated in % (where the value of control animals was 100%).

### 4.18. Determination of the Content of Lipid Peroxidation Products

The level of lipid peroxidation was assessed by determining the content of 2-thiobarbituric acid (TBA) reactive substances. The method is based on the interaction of lipid peroxidation products with TBA to form a colored trimethine complex. A detailed procedure is described in [19].

To 0.2 mL of blood plasma, 3 mL of 10 mM K, Na-phosphate buffer (in 125 mM KCl, pH = 7.4) and 0.5 mL of 1 mM KMnO_4_ were added. To induce lipid peroxidation, 0.5 mL of 10 mM FeSO_4_ was added twice with an interval of 10 min. The reaction was stopped with 1 mL of 20% TCA and samples were centrifuged. To 1 mL of supernatant, 0.25 mL of 1 M HCl and 0.5 mL of 0.7 mM TBA were added and incubated at 100 °C for 20 min. The sample was then cooled, and the light absorption was measured at 532 nm. The results were expressed in nmol per 1 mL of plasma and recalculated in % (where the value of control animals was 100%).

### 4.19. Soluble Lipofuscin Level Assay

For the measurement of the level of soluble lipofuscin, 0.1 mL of plasma was diluted 100-fold in deionized water. The fluorescence spectra of the mixture were measured at excitation wavelengths of 450–650 nm and emission wavelengths of 430 nm [68]. The area under the curve of relative fluorescence was determined in the segment of 492–617 nm, which is characteristic for excitation of lipofuscin. Lipofuscin level was expressed in conventional units (c. u.) per 1 × 10^−6^ g of protein.

### 4.20. Statistical Analysis

Statistical analysis of the results was carried out using Microsoft Excel. To calculate EC_50_ after the DPPH Scavenging Assay, Origin Pro was used. The calculation of statistical parameters was performed by direct quantitative data obtained from the experiment (arithmetic mean—AM; the standard deviation of the arithmetic mean—SD). One-way analysis of variance (ANOVA) with post hoc analysis was used to assess the reliability of the difference between alternative data sets. Pairwise comparisons of data were performed using Tukey’s or Dunnett’s tests. The difference was considered significant at *p* ≥ 0.95 (significance level *p* < 0.05).

All chemical analyses for polyphenolic compound determination were performed six times, and their concentrations were expressed as mean ± standard deviation. Data were subjected to one-way ANOVA for each group of compounds. Then the significance of differences between mean values was calculated using Tukey’s multiple comparison post hoc test at *p* < 0.05.

## 5. Conclusions

This study opens new research prospects for evaluating a natural complex of polyphenols from grape pomace extract on key markers of oxidative stress and metabolic dysregulation in plasma in type 1 diabetes mellitus. Phloretin 2’-glucuronide, peonidin 3-O-glucoside, trans-cinnamic acid, and gallic acid hexoside were found to be the predominant components of grape pomace extract.

The extract exhibited a hypoglycemic effect, evidenced by reduced fasting blood glucose, lower fructosamine concentration, and decreased areas under glycemic curves following glucose and starch loads. Additionally, grape pomace extract improved the lipid profile, normalized paraoxonase activity in the blood plasma of diabetic rats. Treatment of diabetic rats with grape pomace extract enhanced the activities of antioxidant enzymes, accompanied by reduced oxidative modification of proteins and lipids.

Overall, the findings suggest that grape pomace extract modulates oxidative stress, glycemic control, and lipid metabolism through mechanisms likely related to its polyphenolic composition. These results open prospects for further studies on dose–response relationships, determination of the minimal effective dose, identification of potential toxicity thresholds, and elucidation of the molecular targets of polyphenols, to better define the potential of grape pomace extract applications in pharmaceutical and nutraceutical development.

## Figures and Tables

**Table 1:** Concentration of polyphenolic compounds (in mg per mL, *n* = 6) in grape pomace extract.

No	Rt (min)	Compound	Mass Observed ^1^	M	Concentration of Phenolic Compounds
Phenolic acids and derivatives					
1	1.93	Catechol	109.01	110.11	0.190 ± 0.018 a ^2^
2	2.77	Gallic acid	169.00	170.12	0.833 ± 0.022 b
3	4.61	Gallic acid hexoside	331.02	332.26	1.221 ± 0.060 c
4	6.28	Vanillic acid	167.00	168.15	0.214 ± 0.105 a
5	7.14	p-Coumaric acid	163.01	164.16	0.182 ± 0.015 a
6	9.53	trans-Cinnamic acid	147.02	148.16	1.342 ± 0.161 c
Flavonoids and derivatives					
1	5.60	Catechin	289.04	290.27	0.509 ± 0.022 f ^2^
2	6.25	Epicatechin	289.04	290.27	0.637 ± 0.030 g
3	6.97	Rutin	609.11	610.52	0.065 ± 0.011 b
4	7.17	Quercetin 3-O-glucoside	463.06	464.38	0.221 ± 0.007 e
5	7.14	Quercetin 3-O-rhamnoside	447.07	445.38	0.025 ± 0.003 a
6	8.88	Quercetin	301.00	302.24	0.495 ± 0.021 f
7	9.56	Naringenin	271.03	272.25	0.012 ± 0.001 a
8	7.89	Myricetin	317.01	318.24	0.120 ± 0.001 c
9	6.59	Myricetin 3-O-glucoside	479.06	480.38	0.174 ± 0.009 d
Anthocyanins and derivatives					
1	5.95	Malvidin 3-O-glucoside	493.04	493.40	0.995 ± 0.034 c ^2^
2	8.43	Peonidin 3-O-glucoside	463.02	498.90	3.888 ± 0.250 e
3	5.46	Cyanidin 3-O-glucoside	449.08	484.80	0.634 ± 0.027 b
4	6.56	Petunidin 3-O-glucoside	479.23	479.40	0.239 ± 0.039 a
5	7.00	Cyanidin 3-O-(6”-O-acetyl)-glucoside	491.04	491.40	0.724 ± 0.043 b
6	8.98	Delphinidin 3-O-(6”-O-acetyl)-glucoside	507.05	507.40	0.289 ± 0.038 a
7	6.52	Phloretin 2’-glucuronide	437.15	436.40	3.212 ± 0.141 d

^1^ For phenolic acids and flavonoids the observed mass was [M-H]–, whereas for anthocyanins [M + H]+; ^2^ Values for each group of compounds marked by different letters are statistically different at *p* < 0.05 (Tukey’s test).

**Table 2:** Half maximal effective concentration (EC_50_, ug/mL) for grape pomace extract (GPE), Trolox, and Quercitin for DPPH scavenging activity.

Sample	EC_50_ (ug/mL)
GPE	208.27 ± 11.34 ^1^
Trolox	155.96 ± 7.38
Quercetin	77.16 ± 5.08

^1^ Values are arithmetic mean ± standard deviation (*n* = 3).

**Table 3:** Plasma lipid profile in control, diabetes mellitus, and after administration of grape pomace extract.

Experimetal Group	Total Cholesterol, mmol/L	Triglycerides, mmol/L	High-Density Lipoproteins, mmol/L
C	1.31 ± 0.10 ^a^	0.46 ± 0.05 ^c^	1.36 ± 0.07 ^a^
C + GPE	1.57 ± 0.08	0.40 ± 0.03	1.17 ± 0.08
DM	2.79 ± 0.26 ^a^	0.86 ± 0.09 ^c,d^	0.86 ± 0.07 ^a,b^
DM + GPE	1.99 ± 0.29	0.44 ± 0.07 ^d^	1.37 ± 0.06 ^b^

Values are arithmetic mean ± standard deviation (*n* = 6). a, b—data at the column with the same superscript letters are significantly different at *p* < 0.001; c, d—data with the same superscript letters are significantly different at *p* < 0.01.

**Table 4:** The activity of antioxidant enzymes in plasma in control, diabetes mellitus, and after administration of grape pomace extract.

Experimental Group	Superoxide Dismutase, U/ug of Protein	Catalase, nmol H_2_O_2_/min × mg of Protein	Glutathione Peroxidase, umol GSH/min × mg of Protein
C	11.80 ± 0.53 ^a^	3.52 ± 0.29 ^a^	216.23 ± 7.96 ^a^
C + GPE	11.30 ± 0.54	3.11 ± 0.15	218.08 ± 10.08
DM	8.47 ± 0.35 ^a, b^	2.59 ± 0.19 ^a,b^	162.12 ± 9.11 ^a,b^
DM + GPE	12.06 ± 0.79 ^b^	3.83 ± 0.18 ^b^	211.57 ± 7.96 ^b^

Values are arithmetic mean ± standard deviation (*n* = 5). a, b—data at the column with the same superscript letters are significantly different at *p* < 0.05.

## Data Availability

Data are contained within the article.

## Abbreviations

The following abbreviations are used in this manuscript:

AUCglu	area under the glycemic curve
C	control
DM	diabetes mellitus
DPPH	2, 2-diphenyl-1-picrylhydrazyl radical
EC_50_	half-maximal effective concentration
GPE	grape pomace extract
GSH	reduced glutathione
HDL	high-density lipoprotein
LDL	low-density lipoproteins
LPO	lipid peroxidation
NTB	nitrotetrazolium blue
OGTT	oral glucose tolerance test
OSTT	oral starch tolerance test
PUFAs	polyunsaturated fatty acids
RNS	reactive nitrogen species
ROS	reactive oxygen species
TBA	thiobarbituric acid
TCA	trichloroacetic acid
UHPLC-MS	ultra-high performance liquid chromatography coupled mass spectrometry
